# Tapping into the potential of okra (*Abelmoschus* spp.) in Africa: integrating value-added traits into breeding

**DOI:** 10.3389/fpls.2025.1631221

**Published:** 2025-08-14

**Authors:** Mathieu A. T. Ayenan, Fabrice Vihou, Mwasilwa Ambali, Jacinta Adoma Opoku, Dorcas Olubunmi Ibiotye, Roland Schafleitner

**Affiliations:** ^1^ World Vegetable Center, West and Central Africa, Coastal and Humid Regions, Cotonou, Benin; ^2^ Council for Scientific and Industrial Research - Crops Research Institute, Kumasi, Ghana; ^3^ Genetic Resources Unit, National Horticultural Research Institute, Ibadan, Nigeria; ^4^ World Vegetable Center Mexico Office hosted by International Maize and Wheat Improvement Center (CIMMYT), Carretera México-Veracruz, Texcoco, Mexico

**Keywords:** Abelmoschus esculentus, opportunity crops, value-added traits, trait improvement, pectins, mucilage

## Abstract

Okra is a nutritious vegetable of global significance. This crop serves various purposes and presents numerous untapped opportunities. However, several challenges hinder Africa from fully harnessing okra’s economic and nutritional benefits, including pest and disease pressures, salinity and cold stress, weak seed systems, insufficient market knowledge, and a lack of value addition. A wealth of okra genetic resources is conserved in gene banks worldwide; however, only a handful have been characterized for agronomic and value-added traits, limiting their use in breeding programs. Although traditional breeding has focused on enhancing yield, disease resistance, and pod quality traits, which remain important, the growing demand for new okra-based products such as pectin, oil, snacks, and coffee substitutes offers new opportunities for farmers, processors, and breeders. Meeting these demands will require incorporating value-added traits into breeding programs, as identified in this review. Understanding the diversity of okra germplasm for value-added traits, their genetic architecture, and developing efficient screening methods is crucial for creating improved varieties that meet the needs of farmers, processors, and consumers. Enhancing the value-added traits of okra will increase production to meet the rising demand. We identified key traits of interest for improvement across different okra uses. Improving okra for biotic and abiotic challenges, as well as integrating horticultural and value-added traits, requires an in-depth understanding of the okra market to define and prioritize market segments along with target product profiles that fulfill their requirements, increased investment in okra breeding, collaboration between public and private seed and processing firms, resource sharing, and strengthening seed systems. Building capacity in okra breeding and seed technologies is critical to catalyzing okra improvement in Africa.

## 1 Introduction

Okra (*Abelmoschus* spp.) is a highly valued crop cultivated extensively in tropical and subtropical regions, recognized for its nutritional and culinary significance (Elkhalifa et al., 2021). The Asian cultivated species (*A. esculentus* (L.) Moench), also referred to as common okra (or Bhendi in India), is an allotetraploid (2n = 130-140), while the West African cultivated okra, *A. caillei* (A. Chev.) Stevels, is an amphipolyploid (allotetraploid, 2n = 196-200) between *A. esculentus* (2n = 130-140) and *A. manihot* (L.) Medik. (2n = 60-68) (Siemonsma, 1982; Ahiakpa et al., 2017). Okra is predominantly cultivated in Asia and Africa, with production also occurring in Southern Europe and the Americas, underscoring its global significance (Mangan et al., 2008; Sabitha et al., 2011). Okra is mainly grown for its immature pods, which can be consumed fresh or dried and incorporated into various dishes. Although less commonly utilized, okra seeds and leaves are also consumed (Adetuyi and Adelabu, 2011). Immature pods are rich in essential nutrients, including fiber, vitamins A and C, potassium, and iron, while the seeds contain 20% oil and high-quality protein, making them a valuable addition to the human diet (Gemede et al., 2015). Okra is considered a functional food due to its high content of mucilage and bioactive compounds, such as beta-carotene and ascorbic acid, which offer various health benefits (Adetuyi and Adelabu, 2011; Arlai et al., 2012; Ghori et al., 2014; Petropoulos et al., 2018; Dantas et al., 2021). Okra is a nutrient-dense vegetable; for instance, one cup (100 grams) of raw okra contains approximately 36 kilocalories, 8.20 grams of carbohydrates, and 2.10 grams of protein, while being low (0.2 grams) in fat (Adetuyi and Dada, 2014). Okra is exceptionally high in dietary fiber, which aids digestion and helps regulate blood sugar levels. Polyphenols, a type of antioxidant, may enhance okra’s health benefits by reducing oxidative stress and decreasing the risk of chronic diseases (Kendall and Jenkins, 2004; Tseng et al., 2004; Amin and Mahmood, 2011). In addition to its culinary uses, okra holds significant cultural relevance (Woldetsadik et al., 2022). In Nigeria, for instance, the consumption of okra leaves is linked to cultural beliefs about fertility and multiple births (Omonkhua et al., 2020).

Okra is well-suited for warm regions, with an ideal growth temperature of around 30°C, thriving throughout the year in various agro-ecological zones. The significance of okra goes beyond mere subsistence farming; it is vital for crop diversification and income generation for those involved in its value chains (Amin et al., 2023). The okra seed market is a growing sector, with a global value estimated at USD 215.8 million in 2024 and projected to reach USD 380 million by 2030 (Okra Seeds Market Report, 2025). Nonetheless, in Africa, cultivation is limited by minimal investment in breeding improved varieties, the prevalence of farmer seed systems providing seeds of variable quality, and pests and diseases that affect okra globally, resulting in low yields. Additionally, global okra breeding has largely overlooked value addition traits. To address these challenges, it is essential to develop breeding programs that align with consumer preferences, cropping systems, and environmental issues. Current knowledge regarding okra production, its economic significance, and breeding goals in Africa is limited and not well-documented. This lack of comprehensive information hinders understanding of the crop’s importance and the associated challenges and prospects for its improvement. Consolidating and discussing existing information on okra will support investment in improving the crop in Africa. Extensive reviews are focusing on okra breeding (Mishra et al., 2017; Dhankhar and Koundinya, 2020; Mishra et al., 2021; Singh et al., 2023), as well as its uses, properties, and health benefits (Gemede et al., 2015; Dantas et al., 2021; Elkhalifa et al., 2021). This review contributes to the current body of research on okra by summarizing its production and economic value in Africa and examining the breeding needs related to various uses of the crop. Additionally, we mapped existing okra breeding programs and suggested strategies for okra improvement in Africa.

## 2 Cultivated species in Africa

The Asian cultivated species (*A. esculentus*), commonly known as lady’s finger or okra, and referred to as Bhendi in India, has been reported as an amphidiploid with varying chromosome number (2n = 130-140), 2n = 130 being the most frequently reported chromosome number (Singh et al., 2023). Recent genomic information confirmed the allotetraploid nature of *A. esculentus*, with two sub-genomes: one containing 30 chromosomes and another comprising 35 chromosomes (Wang et al., 2023; Nieuwenhuis et al., 2024). For *A. moschata*, a full-length transcriptome has been produced (Guangying et al., 2023). The West African cultivated okra (*A. caillei*) is an amphipolyploid (allotetraploid) with 2n = 194 chromosomes (Singh and Bhatnagar, 1975). This species arose from the cross between *A. esculentus* (2n = 130-140) and *A. manihot* (2n = 60-68) (Siemonsma, 1982). *A. caillei*, or West African okra, is reportedly well-suited to the growing conditions in West and Central Africa, where it is cultivated as a subsistence crop. It is a short-day crop that is sensitive to photoperiodism (Kumar et al., 2010), which limits its adaptability to intensive cultivation conditions. Earliness has become vital for farmers to adapt to their cropping systems and climate change and respond to market demand. The introduction of higher-yielding *A. esculentus* varieties has reduced the land area allocated to *A. caillei*, which is now primarily found in home gardens, accounting for less than 5% of the total okra cultivation area. *A. caillei* has a longer harvest duration and drought tolerance, which can be transferred to *A. esculentus*, an essential trait for okra growers. However, considering its adaptation to growing conditions, beyond using *A. caillei* as a source of traits for *A. esculentus* improvement (Kumar et al., 2010), this species can also benefit from efforts to improve its adaptation to commercial production systems. Gathering market information on consumer acceptability of *A. caillei* will guide decision-making on investing in its improvement. Key traits requiring improvement in *A. caillei* to fit into commercial production systems include photo-insensitivity, earliness, and plant architecture. Additionally, due to its large leaves and extended life cycle, *A. caillei* can be utilized as a dual-purpose crop in communities where both leaves and pods are widely consumed.

## 3 Okra uses and breeding implications

Okra is a multipurpose crop. All the plant parts are used for human consumption or in the non-food industry (**Table 1**). Fresh pods are widely consumed in various forms, such as salads, soups, and stews (Salameh, 2014). The fresh pods are also used for confectionery (Gemede et al., 2015), pickles, and snacks (Gemede et al., 2015). Mucilage is extracted from fresh pods for various uses, including edible films, additives in food formulations, and excipients in the pharmaceutical industry due to its stabilizing, emulsifying, and thickening properties (de Alvarenga Pinto Cotrim et al., 2016; Dantas et al., 2021; Kalkan and Maskan, 2023). Okra pods are amenable to drying to increase storability and reduce shipping costs. Dried okra sauce is widely consumed in West Africa (Kumar et al., 2010). Okra leaves are used fresh or dried for sauces in many communities in West Africa Omonkhua et al., 2020; Borokini et al., 2022). Okra is used in folk medicine. A decoction of the immature capsules is demulcent, diuretic and emollient (Kumar et al., 2013). Okra mucilage has medicinal applications and can be used as a plasma replacement or blood expander due to its ability to increase blood volume (Akinyele and Temikotan, 2007). In Nepal, the juice of the root is used externally to treat cuts, wounds, and boils. It is also considered a suitable coagulant for industrial wastewater treatment (Freitas et al., 2015).

**Table 1:** Okra plant parts and their various uses.

Parts of okra	Uses	References
Fresh pod	• Sauce, soup, salad • Confectionary • Pickle • Mucilage extraction • Snack	Adelakun and Oyelade (2011); Agbo et al. (2010); Adetuyi and Adelabu (2011); Omoniyi et al. (2018); Adetuyi et al. (2008); Gemede et al. (2015); Mohammed et al. (2024); Ofoefule et al. (2001); Kumar et al. (2010); Lengsfeld et al. (2004); Messing et al. (2014); Gemede et al. (2018); Sengkhamparn et al. (2009); Kahlon et al. (2007); Sabitha et al. (2011); (Dantas et al., 2021).
Sliced and dried pod	• Sauce	Adetuyi and Adelabu (2011)
Fresh leave	• Sauce/soup • Salad • Tea	Singh et al. (2014); Borokini et al. (2022); Mohammed et al. (2024)
Dry seed	• Oil • Roasted for coffee substitute (caffeine-free) • Flour for cereal flour fortification	Benchasri (2012); Adelakun et al. (2009); Çalışır et al. (2005); Moekchantuk and Kumar (2004).
Flower	• Tea • Sauce/soup	Klimp et al. (2002); Zheng et al. (2014); Geng et al. (2015); Agbenorhevi et al. (2020).
Stem	• Twin and nets • Firewood • Fiber in the textile industry	Siemonsma (1982); Zandjanakou-Tachin et al. (2021); Gupta and Patra (2021).
Root	• Infusion	Mohammed et al. (2024); Kumar et al. (2010)

Mature okra fruits and stems are utilized in the paper industry, while the roots are used to prepare jaggery (Gupta et al., 2021). Seeds are roasted and ground and used as a substitute for coffee (Moekchantuk and Kumar, 2004; Gupta and Patra, 2021).

While immature okra pods are primarily cooked, there are diverse uses in the food, pharmaceutical, and non-food industries. Okra breeding programs have traditionally focused on improving yield, resistance to pests and disease, and pod quality for cooking with less emphasis on value added traits (Mishra et al., 2021; Singh et al., 2023). Expanding the okra market and tapping into its diverse uses require the integration of value-added traits into breeding objectives.

### 3.1 Fresh pods for food and increased nutrients

Breeding okra for enhanced nutritional quality and yield is essential for addressing food and nutrition security, particularly in sub-Saharan Africa, where mineral deficiencies are prevalent (Alake, 2020). Okra is recognized for its high dietary fiber content and rich profile of essential nutrients, including vitamins and minerals such as calcium, potassium, and magnesium (Gemede et al., 2016; Karmakar et al., 2022). However, anti-nutritional factors like phytic acid can hinder nutrient absorption, necessitating breeding efforts aimed at reducing antinutrient compounds while nutrient levels (Adetuyi and Adelabu, 2011). Genetic improvement of okra can yield varieties with potential to significantly improve the nutritional quality of diets and promote public health (Alake, 2020). Previous research has primarily focused on the genetic diversity underlying the nutritional content of okra in various regions worldwide. For instance, Gerrano (2018) conducted a genetic diversity study based on nutritional traits on 50 accessions obtained from the World Vegetable Center, identifying genotypes VI056457, VI033796, VI060824, VI060802, VI055423, and VI049632 as the most genetically distinct. Similarly, Srivastava et al. (2023) reported that genotypes IC169472 and IC169453 from India exhibited high levels of iodine, crude protein, crude fiber, sodium, and total soluble solids. Kumar et al. (2016) identified genotypes USDO-2546, Punjab Padmini, and Arka Abhay as promising founder lines for breeding high nutritional-quality cultivars. Moreover, Kolawole et al. (2022) identified accessions NHOK-0165, NHOK-0171, NHOK-0188, NHOK-0418, NHOK-0462, NHOK-0544, NHOK-0601, NHOK-0602, NHOK-0622, NHOK-0623, and NHOK-0635 as having desirable agronomic traits along with favorable mineral and proximate composition based on a variability study conducted on 29 okra accessions from the National Horticultural Research Institute genebank in Nigeria. These findings suggest that the genetic diversity within okra germplasm provides a valuable resource for developing cultivars that meet market demands while contributing to improved public health outcomes (Fujimoto et al., 2018). Understanding the genetic architecture of quality traits is crucial for advancing breeding programs (Fayad, 2021). However, there remains a significant gap in research focusing on the genetic control of key biochemical compounds related to okra’s nutritional quality. Furthermore, the influence of environmental factors and agronomic practices on the nutritional content of okra remains to be elucidated.

Varietal differences in protein content have been reported for okra, and this trait can be targeted to enhance the nutritional value of okra (Oyelade et al., 2003). Okra is rich in methionine and tryptophan, making it a good complement to improve the quality of legume- and cereal-based diets. The highest proportion of lipids and proteins in okra is found in the seeds, whether fresh or dry. Understanding whether there is an association between seed content and nutritional content in okra can provide a quick and easy way to phenotype for high-nutrient content. If such an association is established, increasing seed content in okra could increase the crop’s nutritional value. While increased seed content could be desirable for dried and ground okra, consumer acceptability for high seed content, especially where fresh okra pods are used, would need to be understood and factored into the breeding process.

### 3.2 Fresh pods for mucilage extraction

High mucilage content is a must-have trait for okra varieties in West Africa, unlike in many other okra production regions (Kumar et al., 2010; Ahiakpa et al., 2014; Agbenorhevi et al., 2020). The main components of okra mucilage polysaccharides are mannose, rhamnose, glucuronic acid, glucose, arabinose, galacturonic acid, galactose, and xylose (Gao et al., 2018; Wang et al., 2018; Elkhalifa et al., 2021). Breeding programs do not traditionally target high mucilage content (Singh et al., 2023). Besides being a preferred trait in West Africa, powdered high-mucilage okra can be utilized in industrial wastewater treatment as a natural coagulant, removing turbidity, color, and chemical oxygen demand, reducing the environmental impact of industrial processes (Gemede et al., 2018). To address this, breeders should prioritize traits like pod size, mucilage content, and ease of processing, selecting accessions with longer, thicker pods that are rich in mucilage. Few studies have examined variability in okra germplasm collections for mucilage content. Ahiakpa et al. (2014) identified accessions DKA, Amanfrom, Asontem NV, Yeji-Local, and Kortebortor-BAR as having higher mucilage content out of 21 West African okra accessions. (Gemede et al., 2018) reported that accession OPA#7 exhibited the highest mucilage contents in Ethiopia. However, our current knowledge of the genetic architecture of mucilage content in okra is limited. In-depth investigations combining biochemical and genomic approaches are necessary to identify the genetic determinants underlying variations in mucilage rate (Fayad, 2021).

Additionally, it is crucial to assess the environmental influence on mucilage production. The current method for quantifying mucilage content using a viscometer is destructive, time-consuming, and variable, depending on the extraction method and conditions (Ahiakpa et al., 2014; Alba et al., 2015; Agbenorhevi et al., 2020). Extensive screening of germplasm collections and routine phenotyping of breeding materials for this trait will require the development of user-friendly, fast, reliable, and cost-effective methods. Okra mucilage is made up of a complex mixture of polysaccharides, including pectin, cellulose, and hemicelluloses such as xylan and xyloglucan (Sengkhamparn et al., 2009). Pectin is the main polysaccharide responsible for the viscous texture of okra mucilage (Kpodo et al., 2017). The highest mucilage yield (17.65%) and intrinsic viscosity value (11.9 dL/g) were recorded in intermediate-matured okra pods (14–15 days after flowering) and immature pods, respectively (Agbenorhevi et al., 2020). Exploring the possibility of developing a near-infrared reflectance spectroscopy (NIRS) calibration model to screen for mucilage content accurately will improve breeding efficiency for this trait in okra. Near-infrared hyperspectral imaging was used to quantify pectin content in orange peel non-destructively (Badaró et al., 2020), in mulberry fruit (Yang et al., 2021), and to quantify flavonoid content in okra (Cui et al., 2023). The application of near-infrared spectroscopy to quantify pectin content in other crops and compounds in okra suggests that it can be deployed to assess pectin content in okra.

### 3.3 Fresh pods for snack production

Developing okra varieties with market-preferred characteristics is crucial to meet this demand. Mid-sized pods (7 to 10 cm) are particularly well-suited for snack production, as they are smooth and bright green (Abba et al., 2020). Fruit length and pod texture are important traits in okra varieties for adaptation to snack production (Singh et al., 2023). Previous research has identified several promising okra accessions with traits needed for snack production. For instance, Temam et al. (2021) reported accession 29622 as having smooth, green pods. Similarly, Reddy et al. (2016) identified multiple accessions with smooth pod textures, including RNO-201 to RNO-205, RNO-207, RNO-208, RNO-210, RNO-211, RNO-213, and RNO-217 to RNO-220. Regarding fruit length, Binalfew and Alemu (2016) identified accessions 240201-C, 240203-A, 240203-B, 240203-D, 240203-F, 240586-A, 240587-B, and 240592-A as having small fruits. Kumari et al. (2017) further identified the following accessions as possessing small fruit sizes: Pusa Makhamali, VRO-6, Kashi Mohini, Pusa Sawani, Punjab-8, SB-2, 307-10-1, Kashi Satadhari, CO-3, VROB-178, Arka Amanika, IBS-02, Azad Blindi-1, and VRO-106. To date, an okra variety possessing all the desired traits for snack production remains elusive. To address this gap, we propose utilizing the accessions identified in previous studies as parents for hybridization (Yadav et al., 2024). This approach will facilitate the development of hybrids that align with market demands. Additionally, conducting a genome-wide association study (GWAS) for key traits of okra in snack production could help unravel the genetic basis of these traits (Fujimoto et al., 2018).

### 3.4 Seed for oil production

The global demand for vegetable oils is steadily increasing, driven by population growth and the expansion of industrial applications (Hossain et al., 2001; Abba et al., 2020). The cultivation of okra holds potential to significantly contribute to the vegetable oil market, particularly in Asia and Africa. Okra seeds contain comparable edible oil (13.0% to 38.1%) to soybean (17.0%–21.0%), cotton (15.0%–24.0%) and safflower (27.0%–41.0%) (Ergönül and Özbek, 2020; Kammili and Yadav, 2022). Okra oil shares similarities with cottonseed oil, particularly as a good source of unsaturated linoleic acid, which ranges from 32.22% to 43.07% (Liu et al., 2021). The gossypol content in a set of 26 okra accessions was lower (< LOQ-62.459 mg/kg) than the international limit (Kantar et al., 2024), suggesting okra oil is edible. Okra seed is rich in palmitic acid, with the highest value recorded in *A. esculentus* (30.42%), oleic acid (31.885% in *A. manihot)*, and linoleic acid (49.638% in *A. tuberculatus)* (Jarret et al., 2011).

The growing interest in sustainable and nutritious oils highlights the need for breeding programs to improve the oil content and quality of okra seeds. Specific traits of interest in breeding for oil content in okra include seed number per pod, seed yield, and oil content per seed (Zibelo et al., 2016; Kumar et al., 2017; Mohammed et al., 2022) (**Table 2**).

**Table 2:** Key traits of interest for improvement for different okra uses.

Primary uses	Priority traits for breeding
Fresh pod for sauce, soup	Pod length: long, medium, and short, depending on the market Pod color (green to dark green, most popular), red for niche market Pod yield: *short internode, upright branching, number of pods per plant* Resistance to diseases (YVMV, ELCV, other begomovirus diseases) Mucilage content (high for West Africa and low for the rest of Africa) Earliness Spineless Harvest period: a long harvest period for monoculture, a short harvest period when okra is planted as a rotation crop Seed production: High seed setting ability Late pod hardening
Fresh okra pods for mucilage extract	High pod yield High mucilage content* High intrinsic viscosity value* Low seed content
Fresh pod for snack**	Long and tender pod Low mucilage content Low seed content
Sliced and dried pods for powder**	Ease of dehydration Color retention Sensory qualities after drying Increased seed content High dry matter
Oil content**	High oil seed content Low gossypol content and cyclopropenoid fatty acids High seed yield: *Number of pods per plant, number of seeds per pod, seed weight* Dwarf or medium plant height suitable for mechanical harvesting for large-scale production
Coffee substitute**	High seed yield Big seed size Seed shape Higher oil content for aroma Higher fatty acids for flavor

**In addition to agronomic traits, specific traits important for value addition.

*: Higher in non-lignified/immature pods.

Additionally, Kantar et al. (2024) reported several high-oil-content landraces from Turkey, and Jarret et al. (2011) evaluated about 1,100 okra accessions of seven species and found that oil content ranged from 2.51 to 23.2%, with the highest oil content recorded in *A. tuberculatus* accession PI639680 (23%). High oil content was also recorded in accessions PI274350 (21.5%), PI538082 (20.9%), and PI538097 (20.9%), suggesting the potential of the cultivated okra as an oil source (Jarret et al., 2011).

The differences in oil content and profile observed in previous studies (Jarret et al., 2011; Sami et al., 2013; Kantar et al., 2024) provide pieces of evidence to support the development of new cultivars with higher oil content, which could improve the potential of okra as an oil crop. Because okra has not been traditionally grown for its grains, yield evaluation data are limited. However, there are a few studies where grain yield was quantified. There was a wide variation in grain yield, ranging from 3.39 to 5.87 t/ha (Makinde, 2014), 0.368 to 0.684 t/ha (Chattopadhyay et al., 2011), and 0.303 t/ha to 2.3 t/ha (Mohammadi et al., 2016). The reported differences likely depend on growing conditions, genotypes, and agronomic practices.

Overall, a concerted effort to select for traits such as seed quantity, oil percentage, oil functional and nutritional properties is essential in developing new okra varieties that meet consumer preferences and enhance agricultural sustainability and food security. To further advance okra breeding for oil production, a comprehensive characterization of okra germplasm collections for oil content and seed yield is essential to identify accessions with significant oil content potential. Seed oil yield is a complex trait, and a better understanding of its component traits and their genetic architecture is crucial for designing breeding strategies to improve it.

### 3.5 Seed as a coffee substitute

The potential of okra as a coffee substitute, due to its caffeine-free nature, has garnered increasing attention, with several studies highlighting the need for targeted breeding programs to enhance specific traits critical for optimal roasting quality and flavor. Gulsen et al. (2007); Kumar et al. (2023), and Kumar et al. (2010) emphasized the importance of traits such as seed yield, size, shape, and oil content for improving roasting efficiency and flavor profile (**Table 2**). However, current breeding programs have not sufficiently focused on developing varieties tailored to the emerging coffee substitute market (Kumar et al., 2023), indicating a significant research gap that needs to be addressed to meet the growing consumer demand for innovative beverage options.

The success of enhancing okra production through value addition largely relies on processing companies. Value addition in okra will require developing varieties that meet the industry’s specific needs, whether for mucilage extraction, processing, or seed for oil extraction. **Table 2** summarizes key essential traits for value addition in okra. The priority traits identified here should serve as a starting point for a more refined prioritization, depending on the context. The decision to integrate valued-added traits into a breeding pipeline necessitates further prioritization exercises, including market surveys and consultations with okra value chains stakeholders.

## 4 Okra improvement for biotic stresses

Worldwide, one of the most devastating diseases is Yellow Vein Mosaic Disease (YVMD), transmitted by the whitefly (*Bemisia tabaci*), which can cause yield losses ranging from 50% to 100%, depending on the timing of infection during the plant’s growth stages (Jose and Usha, 2003; Fauquet and Stanley, 2005). However, in Africa, Enation Leaf Curl Virus (ELCV) is the most serious viral threat to okra production, with yield penalties of up to 100% (Yadav et al., 2018). In Burkina Faso, a yield loss of up to 55% due to okra leaf curl disease was reported in a local variety, resulting in a financial loss of $11,100 per hectare (Tiendrébéogo et al., 2010b). Insect pests, particularly whiteflies, jassids, and aphids, exacerbate these issues by facilitating viral transmission and directly damaging the plants (Rashida et al., 2005; Kumari et al., 2020). Moreover, root-knot nematodes (*Meloidogyne* spp.), cercosporiose caused by *Cercospora malayensis* and *Pseudocercospora abelmoschii* (Armand et al., 2013), and powdery mildew caused by the air-borne fungus *Erysiphe cichoracearum* (Moharam and Ali, 2012) are known to cause significant damage to okra roots, leading to stunted growth and reduced yields (Suma et al., 2023).

Abang et al. (2014); Abang et al. (2018), and Abang et al. (2024) screened okra accessions for resistance to aphids under various conditions and identified VI033805, VI036213, and VI051114 (Abang et al., 2014), and VI033824 (Abang et al., 2021) as resistant to aphids. *A. esculentus* was overall less attacked than *A. caillei* accessions. The yield of resistant accessions remained very low (less than 2 t/ha) (Abang et al., 2021). Accession VI036213, with a low yield, demonstrated stable resistance to aphids across agroecologies in Central Africa (Abang et al., 2024) and could be a potential donor of aphid resistance.

Jassid infestation on okra was reported in West Africa in 2021, leading to significant yield loss and increased pesticide use by farmers. Putative tolerant okra accessions were identified (Kouadio et al., 2024). However, using these accessions in breeding programs will require further screening and trait development.

Breeding for tolerance to these stresses requires prior knowledge of the spread and diversity of their causal agents across the target regions. However, except for studies on begomovirus (Tiendrébéogo et al., 2010a; Leke et al., 2013) focusing on specific countries, we currently lack information on the diversity of causal agents of the major okra diseases, which is a limitation for accurate screening and breeding. A better understanding of the diversity of causal agents, identification of pest and disease hotspots, and seasonal dynamics of okra pests and diseases will provide valuable information for germplasm screening and selection. Breeding for resistance to biotic stress in okra increasingly focuses on utilizing related *A. esculentus* species that exhibit resistance traits. For instance, *A. caillei* and *A. manihot* are sources of resistance against YVMD (Suma et al., 2023) and ELCV. Integrating advanced breeding techniques, including wide hybridization and molecular approaches (marker-assisted selection, genomic selection), is essential for enhancing genetic diversity and developing resilient okra varieties capable of withstanding these biotic challenges (Singh et al., 2014; Zhan et al., 2019). Wide crosses with *A. esculentus* have been challenging due to fertility issues in F1 due to differences in chromosome number. However, by using *A. esculentus* as a female parent and employing chromosome doubling with colchicine to restore F1 fertility, Suma et al. (2023) have successfully developed interspecific progenies with desirable, essential agronomic traits that have been transferred from related species, thereby broadening the genetic base of cultivated okra.

## 5 Okra improvement for abiotic stress tolerance

*Abelmoschus esculentus* is a warm-season crop adapted to relatively high temperatures. The crop thrives well at temperatures between 25 and 32°C. However, various abiotic stresses, particularly drought, heat, salinity, and cold, negatively affect okra, which significantly impacts its growth and yield. Drought duration and intensity are expected to increase across Africa (Yahaya et al., 2024). Drought stress is a significant concern, as it negatively impacts photosynthesis, resulting in reduced productivity (Asante et al., 2024). The co-occurrence of drought and lower or higher temperature stress further exacerbates these effects, highlighting the need for improved varieties that can withstand these stresses (Asante et al., 2024).

Salinity is a significant abiotic stress that disrupts osmotic balance and ion homeostasis in plants, resulting in stunted growth and yield loss (Ashraf and Harris, 2004). Okra exhibits varying degrees of tolerance to salinity, with specific accessions showing better performance under saline conditions (Haq et al., 2012).

**Figure 1:**
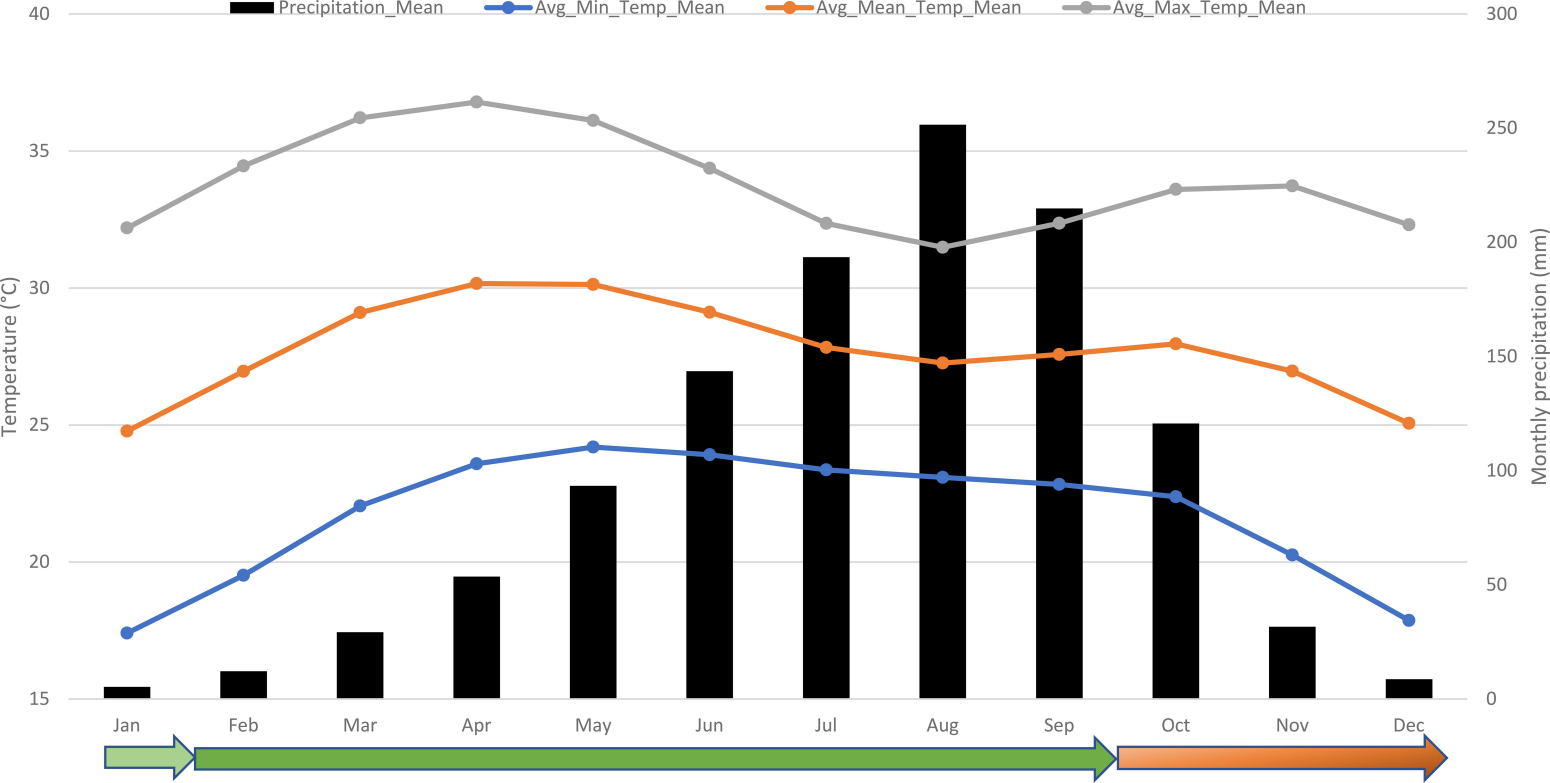
Temperatures and precipitations across West Africa and the okra production seasons in the region. Source: Climate data retrieved from the World Bank Climate Knowledge Portal (https://climateknowledgeportal.worldbank.org/). The color gradient, from light green (moderate suitability) to red (poor suitability), indicates the suitability of the season for okra production.

Okra is sensitive to low temperatures, which can cause chilling injury and hinder growth. Exposure to temperatures below 20°C results in physiological stress in okra, characterized by yellowing leaves and reduced flowering (Omran and Powell, 1971; Huang et al., 2012). Cold stress is significant in the northern regions of coastal and Sahelian countries in West Africa, which account for more than 90% of Africa’s total okra production and harvested area (FAOStat, 2024). Average minimum temperatures (19°C) from November to February are lower than the optimal temperatures for okra growth (**Figure 1**). This period also coincides with a higher incidence of viral diseases. Considering the temperature patterns, February to September is the optimal growing season for okra in West Africa, provided irrigation facilities are available to supply water from February to March-April.

To adapt to abiotic stresses, increased attention and investment should be devoted to developing resilient okra varieties that can thrive under adverse environmental conditions. Given the complex nature of abiotic stress tolerance, investing in trait discovery to identify surrogate traits with less complex genetic architecture for improvement will be critical.

## 6 Seed systems in Africa

The okra seed market in Africa remains underdeveloped, presenting a significant opportunity for growth if the seed sector receives substantial investment and enabling policy and regulatory measures (Kuhlmann et al., 2023). In sub-Saharan Africa (SSA), the okra seed system is primarily informal, with most farmers cultivating local landraces due to cultural preferences and perceived risks associated with new technologies (Nkongho et al., 2022). For instance, in Mali, 14% of okra seeds are locally produced and certified, while the remainder comes from imported seeds and the farmer seed system (Dembélé et al., 2021). Various factors, including limited availability and access to certified seeds—partly due to insufficient investment in local seed production, promotion, and distribution—along with inadequate storage, logistical infrastructure, and policies, impede the availability and use of quality okra seeds, especially in remote areas (Mihretu, 2019; Nkongho et al., 2022). The reliance on seed saving and exchange without quality assurance and control can lead to low seed quality, poor crop establishment, reduced seedling vigor, and lower yields (Hirpa et al., 2010; Nkongho et al., 2022). Additionally, weak extension services and insufficient farmer education exacerbate these challenges, as farmers often lack proper knowledge regarding storage and planting techniques, leading to suboptimal variety performance (Kassie et al., 2015; Nkongho et al., 2022). Okra seed systems in SSA are comparable to some global vegetables, such as tomatoes and peppers, characterized by low local seed production, seed import, and overall high saved seed, exchange, and sourcing from open markets (Ayenan et al., 2021). Okra seed systems hold high potential for development in terms of private sector investment and increased use of improved varieties and high-quality seeds. This is exemplified by the current status of the okra seed sector in Asia especially in India, which is dominated by hybrid varieties (over 80% of the market) mostly developed and marketed by private seed companies (Tikoo, 2025). Addressing okra seed sector’ challenges necessitates a multi-faceted approach that includes investments in seed system development, strengthening extension services, improving infrastructure, and promoting farmer education.

## 7 Diversity of okra germplasm collection

### 7.1 Okra phenotypic diversity assessment in Africa

As of 06 May 2025, approximately 8,000, of which about 5,340 were available for distribution okra accessions, held in various genebanks worldwide, are recorded in the Genesys database (**Table 3**). Knowledge of the existing diversity in these okra collections is crucial for guiding the selection and use of germplasm in breeding programs. We identified 46 studies from Web of Science and Scopus on the characterization and evaluation of okra germplasm, of which 16 were conducted in Africa, reporting the characterization and evaluation of okra accessions under various conditions (**Supplementary Table S1**). In our search, we only included papers published in English, and papers published in languages other than English might have been overlooked. The studies include 160 accessions from Ghana and Nigeria (Annor et al., 2023), 50 accessions (Ibitoye and Kolawole, 2022) and 22 accessions (Kolawole et al., 2022) from Nigeria, 21 accessions (Kenaw et al., 2023) and 33 accessions (Temam et al., 2021) from Ethiopia. All these studies rely solely on phenotypic traits, known to be influenced by environmental factors. Evaluation of okra germplasm for important agronomic and quality traits is even more scarce. Recent studies included germplasm evaluation for mucilage content (Ahiakpa et al., 2014), resistance to okra mosaic virus (OkMV) and Okra yellow vein mosaic virus (OYVMV) (Appiah et al., 2020), drought tolerance (Mkhabela et al., 2022), and resistance to aphids (*Aphis gossypii* Glover) (Abang et al., 2014, 2018, 2021). The characterized accessions represent only a handful of the collected and conserved okra accessions. Characterization of okra germplasm for bioactive compounds is currently scarce.

**Table 3:** Sources of germplasm accessible to okra breeding programs in Africa.

Institutions	Country	Number of accessions in Genesys	Number of accessions in Genesys explicitly indicated as available for distribution (Genesys, GRIN-Global)
USDA	USA	3,289	2,098
World Vegetable Center	Taiwan/Tanzania	2,764	1415
Unité de Génétique, Biotechnologie et Science des Semences	Benin	1,461	1,461
National Center for Genetic Resources and Biotechnology	Nigeria	366	366

From the authors’ discussions with genebank managers (WorldVeg and the University of Abomey-Calavi), more accessions are held in genebanks, but they are yet to be regenerated, characterized and made available on Genesys. Data accessed through GENESYS Global Portal on Plant Genetic Resources, http://www.genesys-pgr.org, and USDA-ARS Germplasm Resources Information Network (GRIN) (https://npgsweb.ars-grin.gov/gringlobal/search) on 06 May 2025. We only included genbanks having okra accessions that were explicitly indicated as available for distribution.

This lack of comprehensive knowledge about the diversity of okra germplasm collections significantly hampers efforts to improve the crop’s productivity and adaptability to various growing conditions (Gulsen et al., 2007; Reddy et al., 2016; Singh et al., 2018; Komolafe et al., 2021; Kumar et al., 2023). Okra faces numerous production constraints, including pest and disease pressures, poor soil fertility, and abiotic stresses (Zibelo et al., 2016; Cobbinah and Kwoseh, 2023). Without a thorough understanding of the genetic diversity within existing germplasm, researchers struggle to identify and utilize sources of important agronomic, adaptation, and quality traits to overcome the numerous biotic and abiotic stresses affecting okra (Kumar et al., 2010). Moreover, limited access to diverse germplasm collections restricts the ability to breed new cultivars with traits such as disease resistance, improved nutritional content, and better tolerance to environmental stresses (Kiiza et al., 2012; Reddy et al., 2016), as well as to identify processing and value-added traits. Addressing these knowledge gaps and expanding the characterization of the okra germplasm collection will significantly enhance the crop’s productivity and utilization.

### 7.2 Molecular diversity of okra germplasm collections

Genetic variation within okra germplasm is crucial for identifying genetically distinct genotypes for hybrid breeding and establishing diversity panels to screen for desirable horticultural and nutritional traits, as well as adaptive mechanisms to breed for tolerance to abiotic and biotic stresses. The genetic diversity of okra germplasm has been extensively studied using various molecular markers (**Supplementary Table S2**), including single nucleotide polymorphism (Sun et al., 2023), random amplified polymorphic DNA (RAPD) (Aladele et al., 2008; Haq et al., 2012; Kaur et al., 2013; Goswami et al., 2016; Hamdan et al., 2024), inter-simple sequence repeats (ISSR) (Yuan et al., 2014; El-Sherbeny et al., 2018), amplified fragment length polymorphism (AFLP) (Akash et al., 2013; Kyriakopoulou et al., 2014; Salameh, 2014; Massucato et al., 2019), sequence-related amplified polymorphism (SRAP) (Gulsen et al., 2007; Yıldız et al., 2016), and simple sequence repeats (SSR) (Sawadogo et al., 2009; Fougat et al., 2015; Yıldız et al., 2015; Kumar et al., 2017; Mohammed et al., 2020; Das et al., 2022) and Isozymes (Torkpo et al., 2006). Most of these studies have focused on a small number of accessions. Additionally, in Africa, very few studies have examined the molecular aspects of diversity. A better understanding of the genetic diversity of the African collection is needed to support the selection of parental lines for breeding programs.

## 8 Prospects for okra breeding in Africa

**Figure 2:**
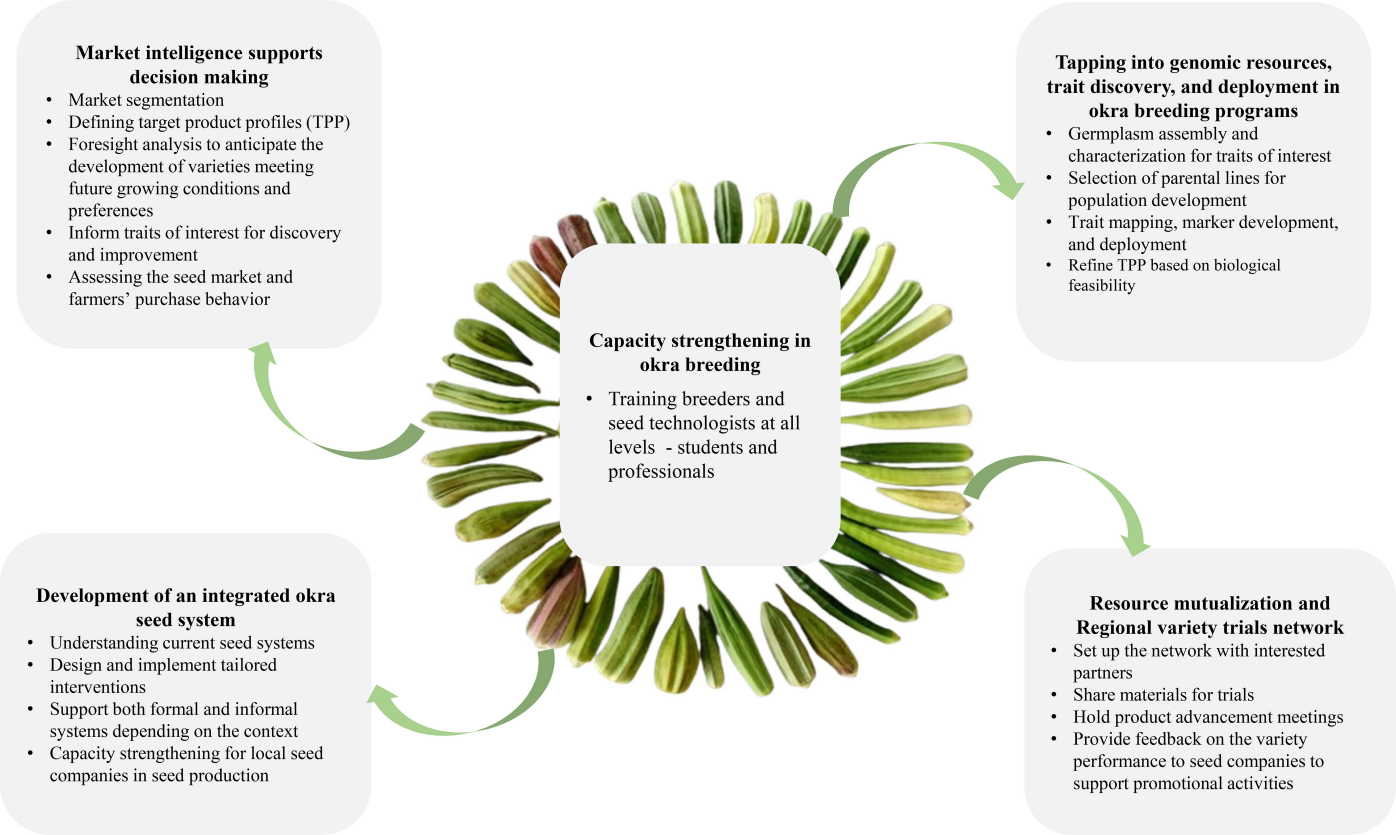
Strategic actions to support okra breeding in Africa.

Enhancing okra breeding programs in Africa necessitates building capacity in okra improvement technologies and methods, collaborating with the private sector to boost investment and adoption of developed varieties, increasing funding for breeding activities, and creating a regional trial network for testing okra varieties. These proposed actions are interconnected, with capacity building at the core of the strategy (**Figure 2**).

### 8.1 Market intelligence for better decision making

Integrating market-driven needs into breeding programs is crucial for effectively deploying improved okra varieties throughout Africa (Tiwari et al., 2022; Singh et al., 2023). Gaining a deeper insight into market requirements is vital for creating competitive and adoptable okra varieties. The market segments for okra in Africa can generally be categorized into long and ridge pod hybrids/OPVs, medium and ridge pods, and short or baby pods. Nonetheless, this broad categorization often fails to acknowledge the significant differences in consumers’ and traders’ preferences, which can vary not only between countries but also within particular regions. Therefore, it is essential to refine the market segmentation. By identifying subtle variations in product preferences and incorporating these preferences as targets in breeding strategies through well-defined, region-specific target product profiles (TPPs), we can considerably enhance the likelihood of variety adoption (Donovan et al., 2022). Varieties that are more closely aligned with consumer expectations, trader needs, and production challenges will experience greater acceptance across the value chain from farmers to markets. This alignment will promote uptake and help farmers and seed companies capitalize on higher-value segments, thereby improving both the competitiveness and profitability of okra production.

### 8.2 Tapping into genomic resources, trait discovery, and deployment in okra

The application of genomics, transcriptomics, metabolomics, and phenomics tools can facilitate our understanding of the genetic foundation of key traits and/or proxy traits such as disease resistance, seed oil quality, and mucilage content, as well as assist in the development and application of markers. Genome-wide association studies (GWAS) are widely used to dissect the genetic basis of complex traits in crops. However, in okra, such studies remain limited due to its complex polyploid genome. Despite this, recent GWAS efforts on okra have identified SNPs associated with important agronomic traits, including seedling tolerance to salinity (Sun et al., 2023), and seed germination under salinity stress (Xu et al., 2024). Schafleitner et al. (2013) generated gene sequence data and marker resources from transcriptomic analyses of leaves and pods in okra. Nevertheless, these genomic resources have not been consistently utilized in okra breeding programs, indicating the need for the creation of customized genomic resources, particularly markers that function effectively without reference sequences and consider not just sequence variation but also copy number polymorphisms (Schafleitner and Lin, 2025). Such genomic resources for marker-assisted selection in okra could be developed through K-mer mapping (Voichek and Weigel, 2020; Schafleitner and Lin, 2025). Overall, there is limited availability of molecular markers for okra breeding due to the complexity of its genome and the absence of a genetic linkage map (Mishra et al., 2017). Despite these challenges, marker-assisted selection (MAS) holds significant promise for accelerating the development of improved okra varieties. Although MAS in okra is still in its early stages, Shwetha et al. (2024) identified molecular markers associated with YVMV resistance in okra, paving the way for early detection, early-stage screening, and the selection of resistant plants.

Whole genome sequences facilitate the development and use of molecular markers such as SNP-markers for genetic analyses. Several whole genome sequences have been produced for okra. A chromosome-scale genome sequence of okra spanning 1.19 Gb over 63 chromosomes was made available (Wang et al., 2023). Another whole genome reference sequence comprises 65 chromosomes and a genome size of 1.45 Gb (Nieuwenhuis et al., 2024). For *A. moschata*, a full-length transcriptome has been produced (Guangying et al., 2023).

The increased availability of genomic information on *A. esculentus* and insight into its genome structure pave the way for genomic-assisted breeding in okra. Marker panels can be developed for quality control and assurance, including verifying varietal identity, testing for genetic purity, and detecting seed lot contamination, thus addressing the gap in this aspect within the public sector. This approach can also benefit small-scale private seed companies. More efforts are needed to sequence and make available the genomes of related species such as *A. caillei*, *A. manihot*, and *A. moschatus*, which serve as donor sources for many traits.

Innovative phenotyping approaches are essential to understand okra response to abiotic stresses and develop resilient okra varieties. High-throughput phenotyping using unmanned aerial vehicles (UAVs) equipped with multispectral cameras offers a significant opportunity to identify novel traits associated with abiotic stress tolerance (Feng et al., 2020; Zhang et al., 2020). While underutilized in okra research, this approach holds considerable promise for trait discovery. Furthermore, understanding the behavior of belowground plant traits is critical for developing drought-tolerant varieties. Root phenotyping can identify promising accessions that exhibit tolerance to abiotic stresses (Wasaya et al., 2018; Canales et al., 2019; Dutta and Sarma, 2022). Integrating aboveground (aerial) and belowground (root) phenotyping provides a comprehensive understanding of plant responses to environmental challenges through trait discovery, ultimately facilitating the breeding of resilient crop varieties (Shi et al., 2023).

In contrast to crops such as tomatoes, where a variety of populations are publicly accessible, this is currently not the case for okra. The development of different population types, including biparental, interspecific, and multi-parent generation inter-cross (MAGIC) populations for specific traits of interest, will facilitate the speed of trait discovery through QTL mapping, marker development, and deployment, as well as product development. Indeed, numerous research teams can utilize these populations for in-depth investigations into the genetic and physiological architecture of important agronomic traits. QTL mapping is a valuable approach for identifying genes associated with agronomic, nutritional, and stress-resistance traits, thereby accelerating crop improvement. However, in polyploid crops, such as okra, QTL mapping is more complex. Recent advances in statistical tools, such as the PolymapR package in R, have enabled effective QTL mapping in allopolyploids. These tools have been successfully applied in other polyploid species, for example, in mapping black spot resistance in climbing rose (Zurn et al., 2018), anthocyanin content in tetraploid roses (Cheng et al., 2021), and canker resistance in Actinidia chinensis kiwifruit, an allohexaploid (Tahir et al., 2020). Their application in okra could facilitate the identification of key genomic regions and support the development of market-preferred, and resilient varieties. Most value-added traits in okra are likely quantitative, hence amenable to genomic selection. To date, there have been no public reports on the application of genomic selection in okra; however, approaches developed in other allopolyploid crops, such as white clover, an allotetraploid (Ehoche et al., 2025), can provide a basis for such an application.

### 8.3 Capacity strengthening in okra breeding

Capacity strengthening is crucial for improving okra breeding in Africa. Analysis of current breeding programs shows a lack of qualified personnel. Enhancing skills for more okra breeders is essential for fostering local efforts. Until enough breeders, seed technologists, and support staff are trained, substantial changes are unlikely. Offering short courses on market segmentation, target product profiles, using okra-related species to enhance traits, marker-assisted selection, experimental design, managing segregating populations, genomic selection, maintaining parental lines, and seed quality control will equip breeders with essential knowledge to enhance their breeding efficiency programs. Internship and research in full-fledged okra breeding programs are good avenues to enhance skills. Current breeding courses across the continent, like the Africa Breeding Academy and other breeding initiatives, offer promising opportunities for capacity building. International agricultural research institutions with breeding programs on okra can also host professionals and students to provide hands-on okra breeding.

### 8.4 Resource mutualization and Regional variety trials network

Resource mutualization is key for success, considering the limited resources available for okra breeding programs. When available, sharing expertise, germplasm, laboratory, and field facilities can enhance okra breeding capabilities on the continent. Access and use of a breeding management system will enhance collaboration and data sharing. However, resource mutualization needs collaboration among institutions. Additionally, better coordination of okra breeding activities could help limit the duplication of resources. Such a coordinate needs a network that offers breeders an exchange platform. A starting point for this network can be a regional variety trial. Regional trials are essential for collecting valuable data to improve breeding efficiency and gathering information on advanced materials for release. Such trials can enable the identification of “regional champion” varieties, which have wide adaptation and could interest seed companies in increasing their market share. The okra breeding programs in Africa are currently too small to bear the cost of regional trials. However, creating a network will minimize trials and variety release costs, since the network of seed companies can take advantage of regionally harmonized seed regulations to release “regional champion” in one country (West Africa) and two countries (Common Market for Eastern and Southern Africa - COMESA, Southern African Development Community - SADC) for seed production and marketing. Such a network will also provide a platform for exchanging materials, knowledge, and valuable market information on okra improvement.

### 8.5 Support the development of an integrated seed system

Seed systems play a crucial role as the primary channels through which farmers obtain high-quality seeds of the crop varieties essential for their production needs (Nkongho et al., 2022). A well-functioning seed system can significantly enhance agricultural productivity by facilitating rapid and cost-effective increases in crop yields. Improving access to high-quality seeds in Africa is paramount for boosting agricultural productivity (Biemond, 2013; Mihretu, 2019). Breeding programs cannot be sustainable unless a vibrant seed system drives the chain. Regulatory and seed policies are in place to support the okra seed system. Increased investment in capacity strengthening for local okra seed production, along with a more conducive environment, for instance, by easing requirements for mandatory variety testing and releasing to locally produce and certify seeds, will favor the development of local and regional seed companies. These companies and seed cooperatives will take up locally bred varieties for seed production and marketing. In the long run, local seed companies can invest in pre-breeding and breeding activities to access new varieties and diversify their portfolio. Seed cooperatives that produce and serve their communities will also be included in seed capacity strengthening activities to ensure that no one is left behind. A more flexible quality assurance scheme can be implemented for these cooperatives to alleviate the burden of the formal seed certification process.

The execution of these strategic initiatives will result in:

- Full-fledged okra breeding programs that use innovative technologies and methods to develop and deliver improved varieties consistently.
- Providing farmers with market-preferred varieties and products, thus improving livelihoods and nutrition.
- Boosting private sector investment in okra breeding and seed systems.
