# Supplementary material for: Tapping into the potential of okra (Abelmoschus spp.) in Africa: integrating value-added traits into breeding
Source: Front Plant Sci. 2025 Aug 14;16:1631221. doi: 10.3389/fpls.2025.1631221 (PMC12391878; doi:10.3389/fpls.2025.1631221)
Supplement: Supplementary file 1 [file Table1.docx]

Supplementary Material

Methodology for the selection of the studies

We searched Web of Science and Scopus in English, without date limitation, using the following search strings:  (“okra OR “Abelmoschus” OR “okro”) AND (characterization” OR “Okra evaluation”) AND (Africa) for the phenotypic characterization and (“okra OR “Abelmoschus” OR “okro”) AND (“genetic diversity” OR “genetic analysis” OR “genetic assessment” OR “molecular characterization”) for the molecular characterization. Papers in English, explicitly addressing the phenotypic characterization of okra in Africa and the molecular characterization of okra worldwide, as indicated by the title and abstract, were retained for full-text reading and data extraction. Papers published in languages other than English were excluded, and potential information on okra characterization may have been overlooked.”

# Supplementary Table 1. Overview of studies characterizing/evaluating okra germplasm collections

| **Type of studies (characterization/evaluation)** | **Number of traits** | **Traits of interest for evaluation studies** | **Outstanding accessions for a given trait** | **Country were the study was conducted** | **References** |
| --- | --- | --- | --- | --- | --- |
| Characterization | 13 |  | DOV-92 and Pusa Bhindi-5 | India | Das, A., Yadav, R.K., Choudhary, H., Lata, S., Singh, S., Kumar, C., et al. (2022). Population structure, gene flow and genetic diversity analyses based on agro-morphological traits and microsatellite markers within cultivated and wild germplasms of okra [Abelmoschus esculentus (L.) Moench.]. *Genetic Resources and Crop Evolution* 69(2)**,** 771-791. doi: 10.1007/s10722-021-01263-9. |
| Evaluation | 29 | Fruit yield, seed yield, and nutrient | 2424446, 240593, 242439, 240581, 242434, 240484, 240598, 240589, and 240584 | Ethiopia | Mohammed, J., Mohammed, W., and Shiferaw, E. (2022). Performance and genetic variability of okra (abelmoschus esculentus (L.) Moench) genotypes in Ethiopia for agromorphology and biochemical traits. *Advances in Agriculture* 2022(1)**,** 5521151. doi: 10.1155/2022/5521151. |
| Characterization | 36 |  |  | Brazil | Silva, E.H.C., Franco, C.A., Candido, W.d.S., and Braz, L.T. (2021). Morphoagronomic characterization and genetic diversity of a Brazilian okra [Abelmoschus esculentus (L.) Moench] panel. *Genetic Resources and Crop Evolution* 68(1)**,** 371-380. doi: 10.1007/s10722-020-00992-7.  Sood, S., Kapoor, D., Devi, J., and Gupta, N. (2017). Multivariate analysis in advance lines of okra (Abelmoschus esculentus). *Indian J Agric Sci* 87**,** 363-368. |
| Evaluation | 12 | Yield and drought tolerance | LS04, LS05, LS06, LS07, LS08, LS10, LS11, LS15, LS18, LS23, LS24, and LS26 | South Africa | Mkhabela, S.S., Shimelis, H., Gerrano, A.S., and Mashilo, J. (2022a). Phenotypic response of okra (Abelmoschus esculentus [L.] Moench) genotypes under drought-stressed and non-stressed conditions. *South African Journal of Botany* 145**,** 293-302. doi: 10.1016/j.sajb.2021.11.008. |
| Evaluation | 68 | Yield, resistance to YVMD, resistance to Shoot and Fruit borer, resistance to Leaf hopper, yield and yield-related traits | EC306731-P, EC305725,IC117175, IC344598, IC433667, IC331214, IC140986, EC305736, EC306737, EC305741, EC305743, EC306724, EC305749, EC306722 EC306706, IC326782, IC433438, IC090262, IC344738, IC344672, IC344599,  IC510728,IC141067 and IC470454 | India | Gangopadhyay, K., Singh, A., Bag, M.K., Ranjan, P., Prasad, T., Roy, A., et al. (2017). Diversity analysis and evaluation of wild Abelmoschus species for agro-morphological traits and major biotic stresses under the north western agro-climatic condition of India. *Genetic resources and crop evolution* 64**,** 775-790. doi: 10.1007/s10722-016-0400-5. |
| Evaluation | 19 | Dietary fibre, Mucilage, iron, Calcium, ascorbic acid, Leaf chlorophyll | Arka Abhay, USDO 2546, Punjab Padmini,  DOV 26, DOV 27, DOV 66, DOV 2, DOV 23, DOV 24, Pusa A 4, P 7. | India | Kumar, R., Yadav, R., Bhardwaj, R., Baranwal, V., Chaudhary, H., and VINOD, V. (2016). Assessment of genetic diversity among okra (Abelmoschus esculentus) genotypes for quality traits. *The Indian Journal of Agricultural Sciences* 86(6)**,** 785-791. |
| Evaluation | 17 | Number of fruit per plant, Number of nodes per plant, Yield per plant. | ‘(IC-169469-1 × PK)-1-2-1-2’ and  ‘(VRH-2 × VRO-6)-9-2-1-3’ | India | Sood, S., Kapoor, D., Devi, J., and Gupta, N. (2017). Multivariate analysis in advanced lines of okra (Abelmoschus esculentus). Indian J Agric Sci 87, 363-368. |
| Evaluation | 32 | Resistance to flood stress | VI033791, VI047518, VI050170 , VI055884 , VI056451, VI059479, VI060132, VI060690B, VI060739A, VI060748A, VI060784, VI060801, VI060806, VI060822, VI060837B, VI060838B, VI060850, VI061719, VI061723, VI061750, VI061803, VI062547 | Taiwan | Schreinemachers, P., Howard, J., Turner, M., Groot, S.N., Dubey, B., Mwadzingeni, L., et al. (2021). Africa’s evolving vegetable seed sector: status, policy options and lessons from Asia. *Food Security* 13(3)**,** 511-523. doi: 10.1007/s12571-021-01146-y. |
| Characterization | 29 |  | LIM, RET, KAR, MES, CLE, EVR, BOY | Greece | Kyriakopoulou, O.G., Arens, P., Pelgrom, K.T., Karapanos, I., Bebeli, P., and Passam, H.C. (2014). Genetic and morphological diversity of okra (Abelmoschus esculentus [L.] Moench.) genotypes and their possible relationships, with particular reference to Greek landraces. *Scientia Horticulturae* 171**,** 58-70. doi: 10.1016/j.scienta.2014.03.029. |
| Evaluation | 10 | Drought and heat tolerance | Perbhani Karanti, IQRA-III, Selection Super Green, Anmol, Line Bourd, Green Gold and OK-1501 | India | Ahmad, I., Rana, R.M., Hassan, M.U., Khan, M.A., and Sajjad, M. (2022). Association mapping for abiotic stress tolerance using heat-and drought-related syntenic markers in okra. *Molecular Biology Reports* 49(12)**,** 11409-11419. doi: 10.1007/s11033-022-07827-x. |
| Evaluation | 15 | Days to 50% flowering, Marketable fruit length, Marketable fruit girth, Pods per plant, Physiological mature pod length, Internode length, Internodes per plant, Physiological mature Pod girth and Pod yield | Punjab, Sabz Pari, F5:6 segregating populations SO8, SO12 and SO15 | India | Khan, M.S., Ali, S., Ali, N., Khan, S.A., Akbar, R., Ansari, M.J., et al. (2023). Response of okra (Abelmoschus esculentus L.) F5: 6 population of for earliness and yield traits. *Pakistan Journal of Botany* 55(2)**,** 689-695. doi: 10.30848/PJB2023-2(26). |
| Evaluation | 13 | Growth habits, leaf, fruit, flower, and seed: color and shape, fruit pubescence | 29622 | Ethiopia | Temam, N., Mohammed, W., and Aklilu, S. (2021). Variability assessment of okra (Abelmoschus esculentus (L.) Moench) genotypes based on their qualitative traits. *International Journal of Agronomy* 2021(1)**,** 6678561. doi: 10.1155/2021/6678561. |
| Evaluation | 10 | Number of branch,  Number of fruit per branch, Yield | 000034 and 000036 | Pakistan | Ali, A., Shah, H., Gul, R., Ahmad, H., Nangyal, H., and Sherwani, K. (2014). Morpho-agronomic characterization of okra (Abelmuscus esculentus L.). *World Applied Sciences Journal* 31(3)**,** 336-340. doi: 10.5829/idosi.wasj.2014.31.03.14317. |
| Characterization | 14 | . | NGB00378b, NGB00297, NGB00347, NGB00293, and NGB00350 | Nigeria | Alake, C.O. (2020). Genetic variability and diversity in okra landraces using agromorphological traits and seed elemental minerals. *International Journal of Vegetable Science* 26(2)**,** 127-149. doi: 10.1080/19315260.2019.1610926. |
| Characterization | 16 | Seed color, leaf rib color, stem color, branching patterns, fruit color, fruit pubescence, number of ridges per fruit. |  | Palestine | Hamdan, Y., and Salameh, A. (2024). Assessing phenotypic diversity between different landraces of okra (Abelmoschus esculentus L.). *Reproduction and Breeding* 4(3)**,** 113-119. doi: 10.1016/j.repbre.2024.04.002. |
| Evaluation | 11 | Yield, inter node distance, number of fruit per plant, fruit weight | Basanthi, Dhanya | India | Mudhalvan, S., and Senthilkumar, N. (2018). Studies on genetic divergence for fruit yield and its component traits in okra [Abelmoschus esculentus (L.) Moench.] genotypes under coastal eco-system. |
| Evaluation | 12 | Leaf area, yield | AE 75, AE 78, AE 36, AE 37, AE 38, AE 39, AE 41, AE 42, AE 43, AE 45, AE 46, AE 47, AE 48, AE 49, AE 50, AE 52, AE 53, AE 54, AE 55, AE 56, AE 58, AE 59, AE 60, AE 61, AE 62, AE 63, AE 64, AE 69, AE 70, AE 71, AE 72, AE 73, AE 74, AE 76, AE 77, AE 79, AE 80, AE 81, AE 83, AE 84, AE 85, AE 86, AE 88, AE 89, AE 90, AE 91, AE 92, AE 93, AE 94,AE 95, AE 96, AE 97 and AE 98 | India | Karthika, N., and Maheswari, T.U. (2019). Genetic divergence studies in bhendi [Ablemoschus esculentus (L.) Moench]. |
| Evaluation | 11 | Yield, thousand seed weight | EC359637 and IARI | India | Ranga, A.D., Chaudhary, A., and Darvhankar, M.S. (2022). Diversity analysis of phenotypic traits in okra (Abelmoschus esculentus L. Moench). *Journal of Horticultural Sciences* 17(1)**,** 63-72. doi: 10.24154/jhs.v17i1.1214. |
| Characterization and Evaluation | 22 | Plant height, Pubescence on stem, Leaf length, Leaf width, Number of days to first flowering,  Number of days to first harvest,  Fruit width, Pubescence on fruit, Number of fruits per plant, Fruit weight, chlorophyll-  α/β,  nutritional conent: Potassium, Zinc, Manganese, Calcium, Sodium | NHOK-0165, NHOK-0171, NHOK-  0188 NHOK-0418, NHOK-0462,NHOK-0544,  NHOK-0601,NHOK-0602, NHOK-0622, NHOK-  0623, and NHOK-0635 | Nigeria | Kolawole, A.O., Ibitoye, D.O., and Aderibigbe, O.R. (2022). Phenotypic diversity and performance of agro-nutritional characters in okra germplasm collection from Nigeria. *Journal of Tropical Agriculture* 60(2). |
| Characterization | 22 |  |  | Nigeria | Komolafe, R.J., Ariyo, O.J., and Alake, C.O. (2021). Diversity in phenotypic traits and mineral elements of Okra (Abelmoschus esculentus L. Moench) genotypes. *International Journal of Agronomy* 2021(1)**,** 5528703. doi: 10.1155/2021/5528703. |
| Characterization | 17 | Plant stem, inflorescence, fruit shape, colour, pubescence, number of ridges per fruit | HSD6, HSD1835, HSD68, HSD21, HSD1348, HSD1349 | Sudan | El Tahir, I.M. (2023). "Phenotypic variations among okra (Abelmoschus esculentus (L.) Moench) genetic resources in Sudan", in: *Genetic Resources*), 20-31. |
| Characterization | 11 |  | IC-9856B, IC331157, IC-342075, IC-332453 and IC-43736 | India | Akotkar, P.K., De, D., and Pal, A. (2010). Genetic variability and diversity in okra (Abelmoschus esculentus L. Moench). *Electronic Journal of Plant Breeding* 1(4)**,** 393-398. |
| Evaluation | 3 | Bhendi yellow vein mosaic disease incidence and severity, yield | DOV-2, DOV-11, DOV-66 | India | Kumar, A., Kumar, M., Kumar, V., Sharma, V.R., and Chaudhary, V. (2020). Genetic divergence studies for yield and quality traits in Okra [Abelmoschus esculentus (L.) Moench.]. *Int J Agricult Stat Sci* 16**,** 355-360. |
| Evaluation | 23 | Yield, number of fruit per plant, fruit length, fruit diameter, Resistance to insect infestation. | NGB00303, NGB00335, and NGB00463 | Nigeria | Olawuyi, O.J., Oyetunde, E.O., Akanmu, A.O., and Olowe, O.M. (2022). "Phenotypic Characterisation of Nine Accessions of Okra (Abelmoschus esculentus (L.) Moench.)," in *Food Security and Safety Volume 2: African Perspectives*. Springer), 389-401. |
| Evaluation | 1 | Resistance to fusarium wilt | 'Santa Cruz-47', 'BR-2399' and 'BR-1449' | Brazil | Aguiar, F.M., Michereff, S.J., Boiteux, L.S., and Reis, A. (2013). Search for sources of resistance to Fusarium wilt (Fusarium oxysporum f. sp. vasinfectum) in okra germplasm. *Crop Breeding and Applied Biotechnology* 13**,** 33-40. doi: 10.1590/S1984-70332013000100004 |
| Characterization | 23 |  |  | Ghana | Oppong-Sekyere, D., Akromah, R., Nyamah, E., Brenya, E., and Yeboah, S. (2020). Morphological characterization of okra (Abelmoschus sp. L.) germplasm in Ghana. *Research and Development in Agricultural Sciences, Book Publisher International***,** 129-146. doi: 10.9734/bpi/rdas/v2. |
| Evaluation | 1 | Resistance to leafhopper | OK-7, OK-9 and Arka Anamika | India | Kadu, R.V., Kulkarni, S.R., Patil, P.V., and Patil, S.K. (2018). Screening of different genotypes of okra [Abelmoschus esculentus (L.) Moench] against leafhopper, Amrasca biguttula biguttula Ishida. *Journal of Entomology and Zoology Studies* 6(5)**,** 1960-1963. |
| Characterization | 30 |  | 245161-A, 240587-A and 245162-B | Ethiopia | Binalfew, T., and Alemu, Y. (2016). Characterization of okra (Abelmoschus esculentus (L.) Moench) germplasms collected from Western Ethiopia. *Int J Res Agric For* 3(2). |
| Evaluation | 1 | Mucilage yield | DKA, Amanfrom, Asontem NV, Yeji-Local and Kortebortor-BAR | Ghana | Ahiakpa, J., Amoatey, H., Amenorpe, G., Apatey, J., Ayeh, E., and Agbemavor, W. (2014). Mucilage Content of 21 accessions of Okra (Abelmoschus spp L.). *Scientia Agriculturae* 2(2)**,** 96-101. doi: 10.15192/PSCP.SA.2014.2.2.96101 |
| Evaluation | 12 | Vitamin C, fruit yield and number of fruit per plant | UEL7 and UEL3 | Brazil | de Araújo, J.P.D., Zeffa, D.M., Spinosa, W.A., Ventura, M.U., Corte, L.E.-D., Gonçalves, L.S., et al. (2021). Evaluation of okra landraces based on agronomic and biochemical traits. *Horticultura Brasileira* 39**,** 223-228. doi: 10.1590/S0102-0536-20210214. |
| Evaluation | 36 | Fruit yield, 100 seed weight, (number of epicalyx per flower, primary branches per plant, mature pod per plant, fruit ridges, internode length |  | Ethiopia | Muluken, D., Wassu, M., and Endale, G. (2016). Variability, heritability and genetic advance in Ethiopian okra [Abelmoschus esculentus (L.) Monech] collections for tender fruit yield and other agro–morphological traits. *Journal of Applied Life Sciences International* 4(1)**,** 1-12. doi: 10.9734/JALSI/2016/19483. |
| Evaluation | 4 | Immature fruit color, fruit pubescence, ridges per fruit and plant height | 9801, Palam Komal, Hisar Unnat, Parbhani Kranti, VRO-6 and VRO4 | India | Samim, S., Sood, S., Singh, A., Verma, A., and Kaur, A. (2018). Morphological characterization of okra [abelmoschus esculentus (L.) moench]. *International Journal of Current Microbiology and Applied Sciences* 7(10)**,** 2011-2019. |
| Evaluation | 13 | Number of days to 50% flowering and number of days to 50% fruiting | Cs-Legon (local accession) and Clemson spineless | Ghana | Amoatey, H., Klu, G., Quartey, E., Doku, H., Sossah, F., Segbefia, M., et al. (2015). Genetic diversity studies in 29 accessions of okra (Abelmoschus spp L.) using 13 quantitative traits. doi: 10.9734/AJEA/2015/ |
| Evaluation | 10 | Leaf axil bearing first fruit, plant height, duration, yield per plant, number of fruits per plant, number of primary branches, fruit weight and fruit length. |  | India | Duggi, S., Magadum, S., Srinivasraghavan, A., Kishor, D., and Oommen, S.K. (2013b). Genetic analysis of yield and yield-attributing characters in okra [Abelmoschus esculentus (L.) Moench]. *Intl. J. Agric. Environ. Biotech* 6(1)**,** 45-50. |
| Evaluation | 2 | Resistance to shoot and fruit borer | Belagavi Local1, Mandya Local3, Mallapalli Local, Nedumangad Local | India | Duggi, S., Magadum, S., Kishor, D., Srinivasraghavan, A., Oommen, S.K., and Arya, K. (2013a). Screening of okra [Abelmoschus esculentus (L.) Moench] genotypes for shoot and fruit borer (Earias vittella Fab.) resistance. *BIOINFOLET-A Quarterly Journal of Life Sciences* 10(2b)**,** 653-657. |
| Characterization | 10 | Yield, number of fruits per plant, fruit weight, fruit girth and number of primary branches |  | India | Shivaramegowda, K.D., Krishnan, A., Jayaramu, Y.K., Kumar, V., and Koh, H.-J. (2016). Genotypic variation among okra (Abelmoschus esculentus (L.) Moench) germplasms in South India. *Plant breeding and biotechnology* 4(2)**,** 234-241. |
| Evaluation |  | Resistance to Bhendi Yellow Vein Mosaic Virus (BYVMV), resistance to whitefly, Resistance to leafhopper, and resistance to shoot and fruit borer. | IC306722, IC90476-1 IC141055, IC141055 and IC140986 | India | Badiger, M., and Yadav, R. (2019). Screening of germplasm of Abelmoschus against biotic stresses. *Indian J. Agric. Sci* 89(12)**,** 2085-2090. |
| Characterization | 13 |  | TR-42-1, TR-77-1 and TR-35-1 | Turkey | Yildiz, M., Sirke, S.T., Koçak, M., Mancak, İ., Özkaya, A.A., Abak, K., et al. (2025). Characterization of a Diverse Okra (Abelmoschus esculentus L. Moench) Germplasm Collection Based on Fruit Quality Traits. *Plants* 14(4)**,** 565. doi: 10.3390/plants14040565. |
| Characterization | 21 |  |  | Turkey | Düzyaman, E. (2005). Phenotypic diversity within a collection of distinct okra (Abelmoschus esculentus) cultivars derived from Turkish land races. *Genetic Resources and Crop Evolution* 52**,** 1019-1030. doi: 10.1007/s10722-004-6118-9. |
| Characterization | 17 |  |  | Brazil | Massucato, L.R., Nakamura, K.K., Ruas, P.M., Zeffa, D.M., Silva, D.J.H.d., and Gonçalves, L.S.A. (2019). Genetic diversity among Brazilian okra landraces detected by morphoagronomic and molecular descriptors. *Acta Scientiarum. Agronomy* 42**,** e43426. doi: 10.4025/actasciagron.v42i1.43426. |
| Characterization | 30 |  |  | Turkey | Yıldız, M., Ekbiç, E., Düzyaman, E., Serçe, S., and Abak, K. (2016). Genetic and phenotypic variation of Turkish Okra (Abelmoschus esculentus L. Moench) accessions and their possible relationship with American, Indian and African germplasms. *Journal of Plant Biochemistry and Biotechnology* 25**,** 234-244. doi: 10.1007/s13562-015-0330-x. |
| Evaluation | 11 | Number of seeds per fruit, Maximum plant height, Days to first flowering, First flowering node,Number of total fruits per plant, First fruit producing node, Fresh fruit weight, Seed yield, 100 seed weight, resistance to okra mosaic virus and leaf curl virus. | KNUST/SL1/07Nkrumahene, DA/08/02Dikaba and GH 5787Asontem, GH6102Fetri and ‘Asontem’ | Ghana | Oppong-Sekyere, D., Akromah, R., Nyamah, E., Brenya, E., and Yeboah, S. (2012). Evaluation of some okra (Abelmoschus spp L.) germplasm in Ghana. *African Journal of Plant Science* 6(5)**,** 166-178. doi: 10.5897/AJPS11.248. |
| Evaluation | 17 | Pod yield | IC282248, IC27826-A, IC29119-B, IC31398-A, IC45732, IC89819, IC89976, IC90107, IC99716 and IC111443 | India | Thirupathi Reddy, M., Hari Babu, K., Ganesh, M., Chandrasekhar Reddy, K., Begum, H., Purushothama Reddy, B., et al. (2012). Genetic variability analysis for the selection of elite genotypes based on pod yield and quality from the germplasm of okra (Abelmoschus esculentus L. Moench). *Journal of Agricultural Technology* 8(2)**,** 639-655. |

We only included papers from Scopus and Web of Science

We categorized as evaluation studies those that purposely selected a germplasm to assess their performance for traits of interest, while the characterization studies were those that primarily aimed at broadly describing a germplasm collection.

# Supplementary Table 2. Overview of molecular markers used in okra germplasm characterization

| **Marker type** | **Number of markers** | **Species** | **Number of genotypes** | **Information on genetic diversity** | **Country where the studies were conducted** | **References** |
| --- | --- | --- | --- | --- | --- | --- |
| SNP | 353,001 | *A. esculentus* | 180 | Three subpopulations with lower differentiation | China | Sun, J., Xu, G., Hu, Y., Cheng, Y., Wang, X., Yang, J., et al. (2023). Genome-wide assessment of genetic diversity and association mapping for salt tolerance traits in okra (Abelmoschus esculentus L. Moench) using genotyping-by-sequencing. *Scientia Horticulturae* 313**,** 111922. doi: 10.1016/j.scienta.2023.111922. |
| SSR | 65 | *A. esculentus* | 96 | Two distinct subpopulations : cultivated and weed | India | Das, A., Yadav, R.K., Choudhary, H., Lata, S., Singh, S., Kumar, C., et al. (2022). Population structure, gene flow and genetic diversity analyses based on agro-morphological traits and microsatellite markers within cultivated and wild germplasms of okra [Abelmoschus esculentus (L.) Moench.]. *Genetic Resources and Crop Evolution* 69(2)**,** 771-791. doi: 10.1007/s10722-021-01263-9. |
| SRAP | 39 | *A. esculentus* | 23 | High genetic relationship among the genotypes | Turkey | Gulsen, O., Karagul, S., and Abak, K. (2007). Diversity and relationships among Turkish okra germplasm by SRAP and phenotypic marker polymorphism. *Biologia* 62(1)**,** 41-45. doi: 10.2478/s11756-007-0010-y. |
| RAPD | 40 | *A. esculentus* | 70 | The genotypes were broadly divided into three clusters. | India | Kaur, K., Pathak, M., Kaur, S., Pathak, D., and Chawla, N. (2013). Assessment of morphological and molecular diversity among okra [Abelmoschus esculentus (L.) Moench.] germplasm. *African Journal of Biotechnology* 12(21). |
| AFLP | - | *A. esculentus* | 50 | Three distinct groups with no overlap between them | India | Kyriakopoulou, O.G., Arens, P., Pelgrom, K.T., Karapanos, I., Bebeli, P., and Passam, H.C. (2014). Genetic and morphological diversity of okra (Abelmoschus esculentus [L.] Moench.) genotypes and their possible relationships, with particular reference to Greek landraces. *Scientia Horticulturae* 171**,** 58-70. doi: 10.1016/j.scienta.2014.03.029. |
| RAPD | 20 | *A. esculentus* | 39 | Seven clusters with similarity ranging from 44.14% to 82.88% | Pakistan | Haq, I.-u.-., Khan, A.A., Khan, I.A., and Azmat, M.A. (2012). Comprehensive screening and selection of okra (Abelmoschus esculentus) germplasm for salinity tolerance at the seedling stage and during plant ontogeny. *Journal of Zhejiang University Science B* 13**,** 533-544. doi: 10.1631/jzus.B1200027. |
| SSR | 19 | *A. esculentus* | 69 | Higher variation among the studied accessions and GAO-5 was found highly diverse and can be exploited for okra improvement | India | Kumar, S., Parekh, M.J., Fougat, R.S., Patel, S.K., Patel, C.B., Kumar, M., et al. (2017). Assessment of genetic diversity among okra genotypes using SSR markers. *Journal of plant biochemistry and biotechnology* 26**,** 172-178. doi: 10.1007/s13562-016-0378-2. |
| RAPD | 27 | *A. esculentus* | 7 | Three subpopulations with similarity ranging from 0.226 to 0.678 | Palestine | Hamdan, Y.A., Hawamda, A.I., Basheer-Salimia, R., and Salman, M. (2024). Genetic diversity assessment of Palestinian okra landraces (Abelmoschus esculentus L.) through RAPD marker. *Genetic Resources and Crop Evolution* 71(7)**,** 3555-3562. doi: 10.1007/s10722-024-01859-x. |
| SSR | 9 | *A. esculentus* | 26 | Three distinct genetic groups with moderate genetic differentiation | South Africa | Mkhabela, S.S., Shimelis, H., Gerrano, A.S., Mashilo, J., and Shayanowako, A. (2022b). Characterization of Okra (Abelmoschus esculentus L.) accessions with variable drought tolerance through simple sequence repeat markers and phenotypic traits. *Diversity* 14(9)**,** 747. doi: 10.3390/d14090747 |
| SSR | 16 | *A. esculentus* | 32 | Three subpopulations with high genetic diversity and population divergence | Ethiopia | Mohammed, W., Amelework, B., and Shimelis, H. (2020). Simple sequence repeat markers revealed genetic divergence and population structure of okra ['Abelmoschus esculentus'] collections of diverse geographic origin. *Australian Journal of Crop Science* 14(7)**,** 1032-1041. |
| RAPD | 14 | *A. esculentus* | 44 |  | India | Prakash, K., Pitchaimuthu, M., and Ravishankar, K. (2011). Assessment of genetic relatedness among okra genotypes [Abelmoschus esculentus (L.) Moench] using RAPD markers. *Electronic Journal of Plant Breeding* 2(1)**,** 80-86. |
| ISSR | 7 | *A. esculentus* | 10 | Two cluster with genetic similarity values varied from 0.714 to 1.00 with an average 0.857 | Egypt | El-Sherbeny, G., Khaled, A., Obiadalla-Ali, H., and Ahmed, A. (2018). ISSR markers linked to agronomic traits in okra. *International Journal of Modern Agriculture* 7(1)**,** 1-7. |
| SRAP | 19 | *A. esculentus* | 60 |  | Turkey | Yıldız, M., Ekbiç, E., Düzyaman, E., Serçe, S., and Abak, K. (2016). Genetic and phenotypic variation of Turkish Okra (Abelmoschus esculentus L. Moench) accessions and their possible relationship with American, Indian and African germplasms. *Journal of Plant Biochemistry and Biotechnology* 25**,** 234-244. doi: 10.1007/s13562-015-0330-x. |
| RAPD | 13 | *A. callei*  *A. esculentus* | 93 | Higher genetic diversity among *A. Esculentus accessions* | Nigeria | Aladele, S.E., Ariyo, O., and De Lapena, R. (2008). Genetic relationships among West African okra (Abelmoschus caillei) and Asian genotypes (Abelmoschus esculentus) using RAPD. *African Journal of Biotechnology* 7(10). |
| RAPD | 22 | *A. esculentus*  *A. ficulneus*  *A. manihot*  *A. moschatus*  *A. tuberculatus* | 260 |  | India | BISHT, I.S., Mahajan, R.K., and Rana, R.S. (1995). Genetic diversity in South Asian okra (Abelmoschus esculentus) germplasm collection. *Annals of applied biology* 126(3)**,** 539-550. doi: 10.1111/j.1744-7348.1995.tb05388.x. |
| SSR | 18 | *A. esculentus*  *A. turberculantus*  *A. manihot*  *A. moschatus* | 24 | Three main clusters at a cut off value of 0.62 | India | Fougat, R.S., Purohit, A.R., Kumar, S., Parekh, M.J., and Kumar, M. (2015). SSR based genetic diversity in Abelmoschus species. *Indian J. Agric. Sci* 85**,** 1223-1228. |
| ISSR | 22 | *A. esculetus* | 24 | The 24 okra accessions were clustered into 4 geographically distinct groups | China | Yuan, C., Zhang, C., Wang, P., Hu, S., Chang, H., Xiao, W., et al. (2014). Genetic diversity analysis of okra (Abelmoschus esculentus L.) by inter-simple sequence repeat (ISSR) markers. *Genet. Mol. Res* 13(2)**,** 3165-3175. doi: 10.4238/2014.April.25.1. |
| Isozyme | 34 | *A. esculentus* | 22 | Three major clusters at the 55% level of similarity | Ghana | Torkpo, S., Danquah, E., Offei, S., and Blay, E. (2006). Esterase, total protein and seed storage protein diversity in Okra (Abelmoschus esculentus L. Moench). *West African Journal of Applied Ecology* 9(1). doi: 10.4314/wajae.v9i1.45677. |
| SSR | 9 | *A. esculentus* | 66 | Two subpopulations with low genetic diversity were found | Turkey | Yıldız, M., Koçak, M., and Baloch, F.S. (2015). Genetic bottlenecks in Turkish okra germplasm and utility of iPBS retrotransposon markers for genetic diversity assessment. *Genetics and Molecular Research*. doi: 10.4238/2015.September.8.20. |
| SSR | 16 | *A. esculentus* | 20 | Five different group were clustered | Burkina Faso | Sawadogo, M., Ouedraogo, J.T., Balma, D., Ouedraogo, M., Gowda, B.S., Botanga, C., et al. (2009). The use of cross species SSR primers to study genetic diversity of okra from Burkina Faso. *African Journal of Biotechnology* 8(11). |
| AFLP | 8 | *A. esculentus* | 21 |  | Jordania | Akash, M.W., Shiyab, S.M., and Saleh, M.I. (2013). Yield and AFLP analyses of inter-landrace variability in okra (Abelmoschus esculentus L.). *Life Science Journal* 10(2)**,** 2771-2779. |
| RAPD | 31 | *A. esculentus*  *A. callai*  *A. manihot*  *A. Moschatus*  *A. tetraphyllus*  *A. ficulneus* | 42 |  |  | Martinello, G.E., Leal, N.R., Amaral Júnior, A.T.d., Pereira, M.G., and Daher, R.F. (2003). Genetic diversity in okra using RAPD markers. *Horticultura Brasileira* 21**,** 20-25. |
| RAPD | 20 | *A. esculentus* | 20 | Four group with similarity value ranged from 0.486 to 0.669 | India | Goswami, A., Singh, B., and Sharma, A. (2016). Analysis of molecular diversity in okra (Abelmoschus esculentus) genotypes using RAPD markers. *The Indian Journal of Agricultural Sciences* 86(10)**,** 1310-1315. |
| AFLP | 8 | *A. esculentus* | 48 |  | Jordanie | Salameh, N.M. (2014). Genetic diversity of okra (Abelmoschus esculentus L.) landraces from different agro-ecological regions revealed by AFLP analysis. *American-Eurasian Journal of Agricultural & Environmental Sciences* 14(2)**,** 155-160. doi: 10.5829/idosi.aejaes.2014.14.02.12289. |
| AFLP | 5 | *A. esculentus* | 30 | Two subpopulations with higher variability | Brazil | Massucato, L.R., Nakamura, K.K., Ruas, P.M., Zeffa, D.M., Silva, D.J.H.d., and Gonçalves, L.S.A. (2019). Genetic diversity among Brazilian okra landraces detected by morphoagronomic and molecular descriptors. *Acta Scientiarum. Agronomy* 42**,** e43426. doi: 10.4025/actasciagron.v42i1.43426. |
